# *N*-(4-Chloro­phen­yl)-2-de­oxy-α-l-ribo­pyran­osylamine

**DOI:** 10.1107/S1600536808011616

**Published:** 2008-06-21

**Authors:** Pei-Hua Shang, Chang-Mei Cheng, Yong-Chong Yang, Ru-Ji Wang, Yu-Fen Zhao

**Affiliations:** a Key Laboratory for Bioorganic Phosphorus Chemistry and Chemical Biology, Ministry of Education, Department of Chemistry, Tsinghua University, Beijing 100084, People’s Republic of China; b Department of Chemistry, Tsinghua University, Beijing 100084, People’s Republic of China

## Abstract

In the crystal structure of the title compound, C_11_H_14_ClNO_3_, inter­molecular hydrogen bonds link mol­ecules in the *ab* plane, forming layers that stack along the *c* axis.

## Related literature

For related literature, see: Durette *et al.* (1978 ▶); Ganem (1966 ▶); Katzen (1979 ▶); Bridiau *et al.* (2007 ▶); Ojala *et al.* (2000 ▶).
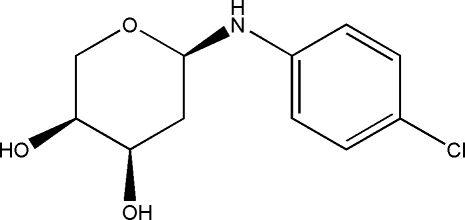


## Experimental

### 

#### Crystal data

C_11_H_14_ClNO_3_
*M* *_r_* = 243.68
Orthorhombic, 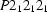

*a* = 6.5305 (8) Å
*b* = 7.9857 (9) Å
*c* = 22.496 (3) Å
*V* = 1173.2 (3) Å^3^
*Z* = 4
Mo *K*α radiation
μ = 0.32 mm^−1^
*T* = 295 (2) K
0.4 × 0.2 × 0.1 mm

#### Data collection

Bruker P4 diffractometer
Absorption correction: none
2581 measured reflections
2172 independent reflections
1690 reflections with *I* > 2σ(*I*)
*R*_int_ = 0.025
3 standard reflections every 97 reflections intensity decay: none

#### Refinement

*R*[*F* ^2^ > 2σ(*F* ^2^)] = 0.037
*wR*(*F* ^2^) = 0.084
*S* = 1.03
2172 reflections
147 parameters
H-atom parameters constrained
Δρ_max_ = 0.15 e Å^−3^
Δρ_min_ = −0.17 e Å^−3^
Absolute structure: Flack (1983 ▶), 880 Friedel pairs
Flack parameter: 0.09 (11)

### 

Data collection: *XSCANS* (Bruker, 1997 ▶); cell refinement: *XSCANS*; data reduction: *XSCANS*; program(s) used to solve structure: *SHELXTL* (Sheldrick, 2008 ▶); program(s) used to refine structure: *SHELXTL*; molecular graphics: *SHELXTL*; software used to prepare material for publication: *SHELXTL*.

## Supplementary Material

Crystal structure: contains datablocks global, I. DOI: 10.1107/S1600536808011616/pk2090sup1.cif

Structure factors: contains datablocks I. DOI: 10.1107/S1600536808011616/pk2090Isup2.hkl

Additional supplementary materials:  crystallographic information; 3D view; checkCIF report

## Figures and Tables

**Table 1:** Hydrogen-bond geometry (Å, °)

*D*—H⋯*A*	*D*—H	H⋯*A*	*D*⋯*A*	*D*—H⋯*A*
O2—H2*C*⋯O3^i^	0.82	1.93	2.739 (2)	170
O3—H3*B*⋯O1^i^	0.82	1.98	2.797 (2)	175
N1—H1*B*⋯O2^ii^	0.92	2.08	2.994 (3)	173

Symmetry codes: (i) 
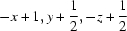
; (ii) 
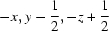
.

**Fig. 1.**
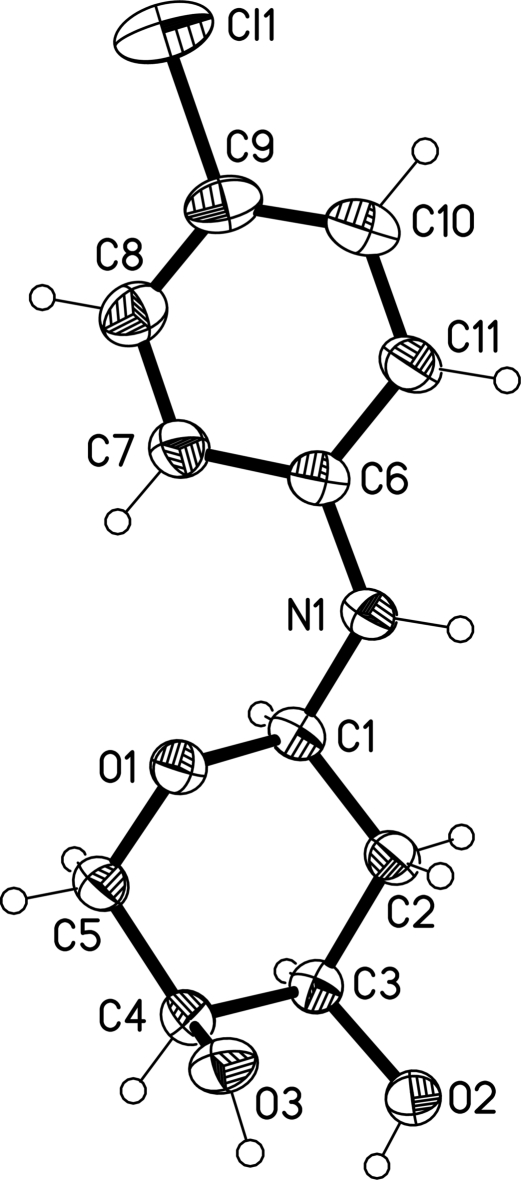
View of the title compound, with displacement ellipsoids drawn at the 35% probability level.

## supplementary crystallographic information

### Comment

*N*-Alkyl and *N*-aryl glycosylamines have a wide range of biological activities (Katzen *et al.*, 1979; Ganem, 1966), including insulin-like activity (Durette *et al.*, 1978). They are important as junctures in glycoproteins (Ojala *et al.*, 2000). Glycosylamines can exist either in cyclic or acyclic forms depending on reaction conditions and the particular amine used.  Stereo-selective syntheses of *N*-aryl-glycosylamines are uncommon, but a one-pot stereoselective synthesis of beta-*N*-aryl-glycosides in aqueous buffers with purification by semi-preparative HPLC has been reported (Nicolas *et al.*, 2007).

Recently, we found that 4-chlorobenzenamine reacted with 2-deoxy-*L*-ribose in methanol and water to give *N*-*p*-chlorophenyl-2-deoxy-α-*L*-ribopyranosylamine as the sole product. Herein we report the synthesis and structure (Fig. 1) of *N*-*p*-chlorophenyl-2-deoxy-α-*L*-ribopyranosylamine.

### Experimental

The title compound was synthesized by the reaction of 4-chlorobenzenamine with 2-deoxy-*L*-ribose in a mixture of methanol and water. 4-chlorobenzenamine (0.93 g, 10 mmol) in a little methanol was added to a solution of 2-deoxy-*L*-ribose (1.34 g, 10 mmol) in 20 ml water, the solution was stirred at room temperature overnight. A white solid obtained by filtration was washed with ice water, then cold ether, and was dried under pressure.  The solid was *N*-*p*-chlorophenyl-2-deoxy-α-*L*-ribopyranosylamine (yield: 70%). ^1^H NMR (300 MHz, DMSO-d_6_): δ 1.68 (m, 1H), 1.78 (m, 1H), 3.37 (d, 1H), 3.49 (s, 1H), 3.62 (q, 1H), 3.68 (m, 1H), 4.36 (d, 1H), 4.56 (m, 1H), 4.69 (d, 1H), 6.16 (d, 1H), 6.53 (d, 2H), 6.886 (d, 2H). ^13^C NMR (300 MHz, DMSO-d_6_): δ 144.2, 129.2, 125.3, 113.4, 80.3, 68.0, 66.8, 65.7, 34.7, 20.1.

### Refinement

H atoms were placed in calculated positions with constrained distances of 0.98 Å (R_3_CH), 0.97 Å (R_2_CH_2_), 0.93 Å (R_2_CH), 0.82 Å (OH) and 0.9195 Å (NH).  U_iso_(H) values were set to 1.2U_eq_ of the attached atom.

### Crystal data

**Table:**

C_11_H_14_ClNO_3_	*F*_000_ = 512
*M**_r_* = 243.68	*D*_x_ = 1.380 Mg m^−^^3^
Orthorhombic, *P*2_1_2_1_2_1_	Mo *K*α radiation λ = 0.71073 Å
Hall symbol: P 2ac 2ab	Cell parameters from 37 reflections
*a* = 6.5305 (8) Å	θ = 4.9–12.5º
*b* = 7.9857 (9) Å	µ = 0.32 mm^−^^1^
*c* = 22.496 (3) Å	*T* = 295 (2) K
*V* = 1173.2 (3) Å^3^	Prism, colorless
*Z* = 4	0.4 × 0.2 × 0.1 mm

### Data collection

**Table:**

Bruker P4 diffractometer	*R*_int_ = 0.026
Radiation source: fine-focus sealed tube	θ_max_ = 25.5º
Monochromator: graphite	θ_min_ = 2.7º
*T* = 295(2) K	*h* = −7→7
ω scans	*k* = −9→9
Absorption correction: none	*l* = −27→27
2581 measured reflections	3 standard reflections
2172 independent reflections	every 97 reflections
1690 reflections with *I* > 2σ(*I*)	intensity decay: none

### Refinement

**Table:**

Refinement on *F*^2^	Hydrogen site location: inferred from neighbouring sites
Least-squares matrix: full	H-atom parameters constrained
*R*[*F*^2^ > 2σ(*F*^2^)] = 0.037	*w* = 1/[σ^2^(*F*_o_^2^) + (0.005*P*)^2^ + 0.4*P*] where *P* = (*F*_o_^2^ + 2*F*_c_^2^)/3
*wR*(*F*^2^) = 0.084	(Δ/σ)_max_ < 0.001
*S* = 1.03	Δρ_max_ = 0.15 e Å^−^^3^
2172 reflections	Δρ_min_ = −0.17 e Å^−^^3^
147 parameters	Extinction correction: none
Primary atom site location: structure-invariant direct methods	Absolute structure: Flack (1983), 880 Friedel pairs
Secondary atom site location: difference Fourier map	Flack parameter: 0.09 (11)

### Special details

**Table:**

Geometry. All e.s.d.'s (except the e.s.d. in the dihedral angle between two l.s. planes) are estimated using the full covariance matrix. The cell e.s.d.'s are taken into account individually in the estimation of e.s.d.'s in distances, angles and torsion angles; correlations between e.s.d.'s in cell parameters are only used when they are defined by crystal symmetry. An approximate (isotropic) treatment of cell e.s.d.'s is used for estimating e.s.d.'s involving l.s. planes.
Refinement. Refinement of *F*^2^ against ALL reflections. The weighted *R*-factor *wR* and goodness of fit *S* are based on *F*^2^, conventional *R*-factors *R* are based on *F*, with *F* set to zero for negative *F*^2^. The threshold expression of *F*^2^ > σ(*F*^2^) is used only for calculating *R*-factors(gt) *etc*. and is not relevant to the choice of reflections for refinement. *R*-factors based on *F*^2^ are statistically about twice as large as those based on *F*, and *R*- factors based on ALL data will be even larger.

### Fractional atomic coordinates and isotropic or equivalent isotropic displacement parameters (Å^2^)

**Table:**

	*x*	*y*	*z*	*U*_iso_*/*U*_eq_	
Cl1	0.01956 (18)	−0.12260 (10)	0.00675 (4)	0.0884 (3)	
O1	0.3998 (2)	0.56562 (19)	0.16726 (7)	0.0425 (4)	
O2	0.2117 (3)	1.0211 (2)	0.23361 (8)	0.0482 (5)	
H2C	0.2839	1.1049	0.2316	0.058*	
O3	0.5280 (3)	0.7894 (2)	0.25924 (7)	0.0477 (4)	
H3B	0.5420	0.8705	0.2812	0.057*	
N1	0.0604 (3)	0.4754 (3)	0.16049 (9)	0.0495 (6)	
H1B	−0.0305	0.4823	0.1916	0.059*	
C1	0.1909 (4)	0.6147 (3)	0.15314 (11)	0.0412 (6)	
H1A	0.1850	0.6517	0.1116	0.049*	
C2	0.1280 (4)	0.7577 (3)	0.19294 (12)	0.0457 (6)	
H2A	0.1197	0.7179	0.2336	0.055*	
H2B	−0.0071	0.7960	0.1813	0.055*	
C3	0.2759 (4)	0.9032 (3)	0.19013 (10)	0.0392 (6)	
H3A	0.2653	0.9554	0.1508	0.047*	
C4	0.4948 (4)	0.8459 (3)	0.19940 (10)	0.0412 (6)	
H4A	0.5884	0.9386	0.1906	0.049*	
C5	0.5384 (4)	0.7017 (3)	0.15789 (11)	0.0451 (6)	
H5A	0.5270	0.7400	0.1171	0.054*	
H5B	0.6776	0.6631	0.1641	0.054*	
C6	0.0505 (4)	0.3402 (3)	0.12239 (11)	0.0450 (6)	
C7	0.2014 (5)	0.3069 (4)	0.08000 (12)	0.0549 (7)	
H7A	0.3118	0.3797	0.0761	0.066*	
C8	0.1893 (5)	0.1674 (4)	0.04379 (12)	0.0591 (8)	
H8A	0.2906	0.1472	0.0156	0.071*	
C9	0.0287 (6)	0.0594 (3)	0.04936 (11)	0.0567 (8)	
C10	−0.1245 (5)	0.0904 (4)	0.08970 (12)	0.0580 (8)	
H10A	−0.2348	0.0172	0.0928	0.070*	
C11	−0.1145 (5)	0.2305 (3)	0.12566 (12)	0.0527 (7)	
H11A	−0.2198	0.2518	0.1525	0.063*	

### Atomic displacement parameters (Å^2^)

**Table:**

	*U*^11^	*U*^22^	*U*^33^	*U*^12^	*U*^13^	*U*^23^
Cl1	0.1371 (9)	0.0575 (4)	0.0707 (5)	0.0048 (6)	−0.0236 (6)	−0.0194 (4)
O1	0.0405 (9)	0.0404 (9)	0.0464 (9)	−0.0016 (8)	−0.0010 (8)	−0.0021 (8)
O2	0.0471 (11)	0.0378 (10)	0.0595 (10)	−0.0028 (9)	0.0080 (9)	−0.0056 (9)
O3	0.0561 (11)	0.0442 (9)	0.0429 (9)	0.0035 (10)	−0.0106 (9)	−0.0058 (8)
N1	0.0488 (13)	0.0482 (12)	0.0515 (12)	−0.0157 (11)	0.0108 (11)	−0.0106 (11)
C1	0.0396 (13)	0.0424 (13)	0.0417 (13)	−0.0043 (12)	−0.0045 (11)	0.0018 (12)
C2	0.0380 (14)	0.0423 (14)	0.0568 (15)	−0.0025 (11)	−0.0008 (12)	−0.0025 (13)
C3	0.0419 (14)	0.0359 (13)	0.0399 (12)	0.0022 (11)	−0.0002 (11)	0.0037 (11)
C4	0.0392 (14)	0.0423 (13)	0.0422 (12)	−0.0042 (12)	0.0011 (11)	0.0017 (10)
C5	0.0415 (14)	0.0480 (13)	0.0457 (13)	−0.0077 (13)	0.0025 (12)	−0.0025 (12)
C6	0.0501 (15)	0.0422 (13)	0.0427 (13)	−0.0043 (13)	−0.0031 (12)	0.0007 (11)
C7	0.0566 (17)	0.0569 (17)	0.0513 (15)	−0.0107 (16)	0.0078 (15)	−0.0060 (14)
C8	0.073 (2)	0.0582 (18)	0.0464 (15)	0.0010 (18)	0.0044 (15)	−0.0038 (14)
C9	0.085 (2)	0.0446 (14)	0.0408 (13)	0.0014 (17)	−0.0127 (16)	−0.0011 (12)
C10	0.0685 (19)	0.0525 (17)	0.0529 (16)	−0.0179 (16)	−0.0067 (15)	0.0033 (15)
C11	0.0533 (16)	0.0551 (17)	0.0497 (15)	−0.0173 (15)	0.0028 (14)	−0.0034 (14)

### Geometric parameters (Å, °)

**Table:**

Cl1—C9	1.742 (3)	C3—H3A	0.9800
O1—C5	1.430 (3)	C4—C5	1.510 (3)
O1—C1	1.454 (3)	C4—H4A	0.9800
O2—C3	1.421 (3)	C5—H5A	0.9700
O2—H2C	0.8200	C5—H5B	0.9700
O3—C4	1.436 (3)	C6—C11	1.391 (4)
O3—H3B	0.8200	C6—C7	1.397 (4)
N1—C6	1.380 (3)	C7—C8	1.382 (4)
N1—C1	1.411 (3)	C7—H7A	0.9300
N1—H1B	0.9195	C8—C9	1.364 (4)
C1—C2	1.508 (3)	C8—H8A	0.9300
C1—H1A	0.9800	C9—C10	1.373 (4)
C2—C3	1.512 (3)	C10—C11	1.382 (4)
C2—H2A	0.9700	C10—H10A	0.9300
C2—H2B	0.9700	C11—H11A	0.9300
C3—C4	1.515 (3)		
			
C5—O1—C1	110.91 (18)	O3—C4—H4A	109.4
C3—O2—H2C	109.5	C5—C4—H4A	109.4
C4—O3—H3B	109.5	C3—C4—H4A	109.4
C6—N1—C1	124.9 (2)	O1—C5—C4	111.7 (2)
C6—N1—H1B	119.3	O1—C5—H5A	109.3
C1—N1—H1B	115.6	C4—C5—H5A	109.3
N1—C1—O1	109.19 (19)	O1—C5—H5B	109.3
N1—C1—C2	111.3 (2)	C4—C5—H5B	109.3
O1—C1—C2	109.23 (19)	H5A—C5—H5B	107.9
N1—C1—H1A	109.0	N1—C6—C11	119.7 (2)
O1—C1—H1A	109.0	N1—C6—C7	122.7 (2)
C2—C1—H1A	109.0	C11—C6—C7	117.6 (2)
C1—C2—C3	112.6 (2)	C8—C7—C6	121.0 (3)
C1—C2—H2A	109.1	C8—C7—H7A	119.5
C3—C2—H2A	109.1	C6—C7—H7A	119.5
C1—C2—H2B	109.1	C9—C8—C7	120.0 (3)
C3—C2—H2B	109.1	C9—C8—H8A	120.0
H2A—C2—H2B	107.8	C7—C8—H8A	120.0
O2—C3—C2	107.00 (19)	C8—C9—C10	120.5 (3)
O2—C3—C4	112.6 (2)	C8—C9—Cl1	120.2 (2)
C2—C3—C4	111.4 (2)	C10—C9—Cl1	119.3 (2)
O2—C3—H3A	108.6	C9—C10—C11	119.9 (3)
C2—C3—H3A	108.6	C9—C10—H10A	120.1
C4—C3—H3A	108.6	C11—C10—H10A	120.1
O3—C4—C5	108.14 (19)	C10—C11—C6	121.0 (3)
O3—C4—C3	111.5 (2)	C10—C11—H11A	119.5
C5—C4—C3	108.8 (2)	C6—C11—H11A	119.5

### Hydrogen-bond geometry (Å, °)

**Table:**

*D*—H···*A*	*D*—H	H···*A*	*D*···*A*	*D*—H···*A*
O2—H2C···O3^i^	0.82	1.93	2.739 (2)	170
O3—H3B···O1^i^	0.82	1.98	2.797 (2)	175
N1—H1B···O2^ii^	0.92	2.08	2.994 (3)	173

Symmetry codes: (i) −*x*+1, *y*+1/2, −*z*+1/2; (ii) −*x*, *y*−1/2, −*z*+1/2.
